# Rheumatoid Arthritis, Kartagener's Syndrome, and Hyperprolactinemia: Who Started It?

**DOI:** 10.1155/2016/7367232

**Published:** 2016-01-20

**Authors:** Hussein Halabi, Israa Mulla

**Affiliations:** King Faisal Specialist Hospital & Research Center, P.O. Box 40047, Jeddah 21499, Saudi Arabia

## Abstract

We report a case of an 18-year-old girl who presented to our hospital with history of recurrent respiratory infections, amenorrhea, and symmetric polyarthritis. She was diagnosed with rheumatoid arthritis (RA), Kartagener's syndrome (KS), and hyperprolactinemia. There have been very few case reports in the literature of RA occurring in the setting of KS, theoretically proposed to be due to chronic stimulation of the immune system by recurrent infections. Furthermore, hyperprolactinemia has been hypothesized to mirror RA disease activity and case reports of treatment with dopamine agonists have led to the speculation of whether or not they represent a new line of experimental treatment in the future. Our patient was found to have both KS and hyperprolactinemia together in the setting of RA, and based on our literature search, this is the first reported case of such a combination. This strikes a very intriguing question: are these three conditions interlinked by a yet to be defined association? And treatment of which condition leads to the resolution of the other?

## 1. Introduction

It has been postulated that recurrent bacterial and viral infections are one of the contributing factors to developing autoimmunity in RA, mainly through stimulation of Toll-like receptors expressed in synovial tissue [1]. This in turn will lead to release of various cytokines and inflammatory mediators with constant triggering of the innate immunity, and when this occurs in a genetically susceptible individual, RA is thought to eventually develop. This has led researchers to question whether KS is somehow linked to developing RA. There are nine case reports in the literature of RA and KS occurring together but what's interesting and different about our case is that our patient also had hyperprolactinemia, which up to our knowledge did not occur in any other reported case [2–10]. Prolactin has also been hypothesized as a possible immune stimulatory modulator in RA but its definite role still remains unclear for studies have yielded inconsistent results [11–13]. We describe our case and summarize the previous case reports and their findings including HLA types to help outline a clear association in the future.

## 2. Case Report

An 18-year-old girl with history of chronic sinusitis and repeated childhood chest infections was referred to our hospital for work up of a nonresolving pneumonia.

**Figure 1:**
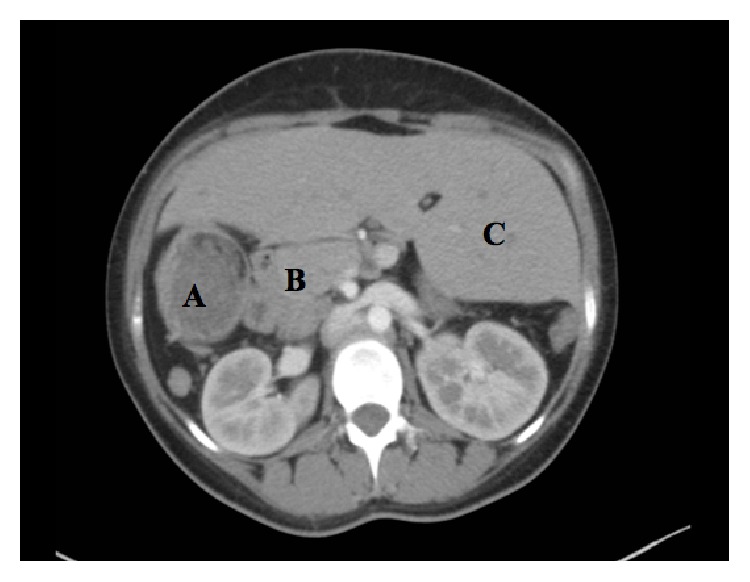
CT scan of the chest with contrast demonstrating situs ambiguous: (A) a right-sided stomach, (B) multiple splenules on the right, and (C) the liver in the midline occupying both right and left portions of the abdomen.

The patient was first seen and evaluated in Pulmonology Clinic. She had a chronic productive cough but no fever, weight loss, or hemoptysis. Chest X-ray revealed a residual right middle lobe infiltrate with no pleural effusion. Computed tomography (CT) scan of the chest revealed patchy airspace opacities in the right lower lobe associated with bilateral lower lobe bronchiectatic changes. Interestingly, there were multiple structural abnormalities incidentally found in the form of an abdominal situs ambiguous with the liver seen in the midline, a right-sided stomach, and multiple small splenules in the right upper abdomen (Figure 1). Bronchoscopy with bronchoalveolar lavage was positive for* Haemophilus influenzae*. All other differentials including* Mycobacterium tuberculosis*, fungal infections, and malignancy were ruled out.

During that same period, the patient gave history of two new complaints: joint pain and swelling and amenorrhea. Regarding her joint complaints, the patient gave history typical of RA with symmetrical joint pain and swelling involving her small and large joints with early morning stiffness of more than an hour. On examination her tender joint count (TJC) was 10 and swollen joint count (SJC) was 7. She had a strongly positive Rheumatoid Factor of 350 and an anti-cyclic citrullinated peptide (Anti-CCP) of 133. Her erythrocyte sedimentation rate (ESR) and C-reactive protein (CRP) were mildly elevated at 21 and 5.27 mg/L, respectively. The rest of her autoimmune panel including ANA and ENA profiles was negative. X-rays of the hands, elbows, shoulders, knees, and feet bilaterally revealed maintained joint spaces and no evidence of erosions. Based on these findings the patient was diagnosed to have RA. Her calculated DAS-28 score was 5.77, her Simple Disease Activity Index (SDAI) 32.5, and her Clinical Disease Activity Index (CDAI) 32, all reflecting high disease activity. She was started on Methotrexate at a dose of 12.5 mg orally once weekly with supplemental folate. She was also kept on Prednisolone 10 mg orally daily with calcium and vitamin D.

As for her amenorrhea symptoms, the patient had irregular periods after menarche but became completely amenorrheic around 8 months prior to presentation. She had been married for one and a half years, had no children, and was not using any method of contraception. She was referred to Obstetrics and Gynecology Team for further evaluation and her work-up revealed the presence of a very high prolactin level of 4425 *μ*g/L (normal range: 3–24 *μ*g/L). another hormonal panel was within normal. She had no symptoms of galactorrhea, was not taking any new drugs, and had no history of renal, thyroid, or liver disease. She had no visual symptoms or headache or vomiting. Pelvic ultrasound showed a retroverted uterus with no abnormalities. She was given an appointment to do pituitary magnetic resonance imaging (MRI) in addition to Endocrinology followup.

**Figure 2:**
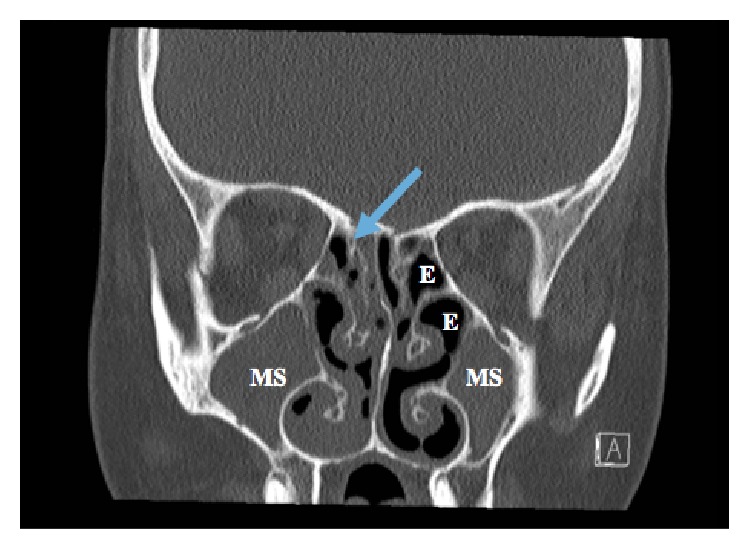
CT scan of the paranasal sinuses showing complete opacification of the maxillary sinuses (MS) bilaterally and some of the ethmoid air cells (arrow) including the sphenoid sinuses. There is also obliteration of the osteomeatal complexes bilaterally.

**Figure 3:**
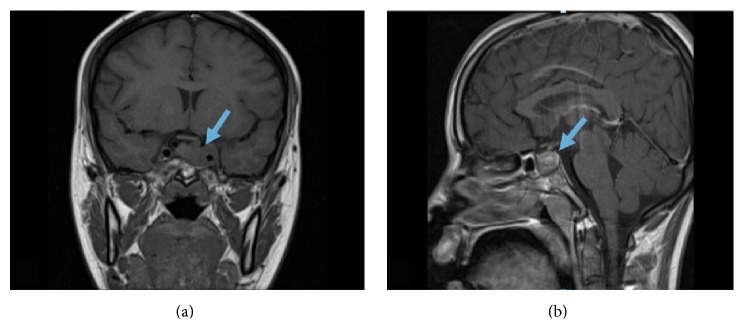
T1 coronal (a) and T1 sagittal (b) sections of the brain on presentation showing a 2.2 × 2.1 × 2.2 cm intrasellar mass in keeping with a pituitary macroadenoma invading the left cavernous sinus and completely encasing the left internal carotid artery.

Due to the earlier CT findings of situs ambiguous and history of chronic bronchiectasis, the diagnosis of KS was entertained. CT scan of the paranasal sinuses was therefore requested and it confirmed the presence of pansinusitis with complete opacification of the maxillary, sphenoid, and ethmoid sinuses (Figure 2). Based on the presence of this triad, she was diagnosed with KS and was admitted for full work-up and treatment on the 20th of October 2014. Magnetic resonance imaging (MRI) of the pituitary gland was done and it revealed a 2.2 × 2.1 × 1.2 cm intrasellar pituitary macroadenoma (Figure 3). She was seen and evaluated by Endocrinology Team and started on Cabergoline 0.5 mg once weekly and discharged in a stable condition with outpatient followup.

When the patient presented to Rheumatology Clinic the following month, she reported almost 80% improvement of her symptoms with significant reduction in her joint pain and complete resolution of morning stiffness and fatigue. Based on assessment that day, her DAS-28 score was 1.7 and her SDAI and CDAI scores were 4.9 and 3, respectively. Her prolactin level dropped to 293 *μ*g/L. The patient achieved clinical remission and her Methotrexate dose was increased to 15 mg orally once weekly. She was instructed to taper down her steroids until tapering off. During the next visit three months later, she was still in remission with a DAS-28 score of 1.4, SDAI of 2.5, and CDAI of 2. Her Methotrexate dose was maximized to 20 mg once weekly. Repeated MRI of the pituitary gland revealed regression in the size of the macroadenoma to 1.4 × 1.4 cm with a further drop in her prolactin level to 28.9 *μ*g/L. Cabergoline was increased to 1.5 mg orally twice a week on subsequent Endocrinology followup.

## 3. Case Discussion

RA is a chronic disabling autoimmune disorder, affecting people from all around the world. The annual incidence is around 40 per 100,000 persons but the prevalence varies according to ethnical background, estimated to be around 1% in North Americans [14]. Many theories have been postulated in the pathogenesis of RA but experts generally agree that it is a result of a complex interaction between environmental and genetic factors in an already susceptible individual [15]. Genetic susceptibility to RA has been extensively studied and still remains an area for burning research. Many genes have been identified as conferring a risk, most importantly the HLA Major Histocompatibility Complex (MHC) Genes, namely, the HLA-DRB1 gene and the DR4 family of alleles. This in addition to the theory of the shared epitope increases risk of severe erosive disease.

However, genes, although an essential part of the pathogenesis, are not the lone culprits, and other environmental factors play a crucial and influential role in triggering RA. Of these, the most well studied and affirmed is smoking, which can interact with the shared epitope, increasing the risk of developing RA up to 40-fold. Another important and established factor is repeated infection. This will lead to recurrent activation of the immune system ultimately leading to an autoimmune state that gradually ensues. As a matter of fact, in one of the earlier reports of RA and KS occurring concurrently together, Beutler et al. hypothesized this very notion, claiming that it was in fact the repetitive triggering of the immune system by chronic sinusitis and bronchiectasis in patients with KS that eventually led to the unveiling of RA. But if so, does treatment of infection lead to resolution of synovitis and subsequent remission? Of the nine published case reports so far linking RA to KS, and excluding those published in a language other than English, all patients except one presented with erosive disease (Table 1). There has been great emphasis over the past couple of years on early initiation of disease-modifying antirheumatic drugs as soon as a diagnosis of RA is established and delaying RA treatment to test this theory in patients who already have erosions is not always feasible [16]. Our patient had no erosions on X-rays but she had clinically significant synovitis with multiple tender and swollen joints. She was initiated on Methotrexate in addition to steroids and when she presented to the clinic three months after, she was in complete remission with a DAS-28 score of 1.7, SDAI of 4.9, and CDAI of 3. Based on this, it is safe to conclude that treatment with disease-modifying antirheumatic drugs is of utter importance and still remains the cornerstone of therapy.

KS is classically known to present with a triad of chronic sinusitis, bronchiectasis, and situs inversus. Genetically speaking, there has not yet been a clear link between RA and Kartagener's syndrome in terms of a susceptibility gene causatively leading a patient with one condition to develop the other. On reviewing the patients reported in the nine cases aforesaid, and excluding four articles that were not in English, three had undetermined HLA status and two had HLA types not known to be associated with RA (Table 1). Based on these limited results, it is impossible to establish a real connection and data is still insufficient to really prove anything.

What makes our case even more interesting is that our patient had hyperprolactinemia in addition to RA and KS. Based on literature review, this is the first case linking all three. Many studies have been conducted fervently evaluating the role of prolactin and the neuroendocrine system in promoting autoimmunity. Some studies, on the other hand, have claimed a cartilage protective effect. Case reports of experimental treatment with dopamine agonists such as Bromocriptine have also been inconsistent, in some showing improvement in the in vitro immune function and HAQ disability scores, whereas in others exhibiting nonsignificant improvement as compared with placebo [17, 18]. Further studies are definitely warranted to outline the exact role of prolactin in association with autoimmunity but until then its influence still remains elusive.

Our patient was treated with Cabergoline, a dopamine agonist, for her prolactin-secreting macroadenoma and she responded remarkably to treatment with a drop in her prolactin level from 4425 to 293 *μ*g/L when she first achieved clinical remission of RA. But does this drop merely signify response to treatment or does it, in theory, mirror her RA disease activity? Interestingly, when the patient's prolactin dropped further to 28.9 *μ*g/L, her inflammatory markers also dropped (ESR to 5 and CRP to 2.14 mg/L). Moreover, if in fact prolactin does stimulate autoimmunity and RA as proposed by some theories, was the presence of hyperprolactinemia a triggering factor to development of RA or was their concurrence together just a sheer coincidence? Our patient was treated for all of her medical problems so it is difficult to conclude if a specific treatment led to resolution of the other condition(s) or if treatments of everything worked synergistically together.

In summary, we report a case of an 18-year-old girl who presented with RA, KS, and hyperprolactinemia. She was successfully treated with a combination of Methotrexate and Cabergoline. Her macroadenoma regressed in size, her bronchiectasis was treated, and she achieved clinical remission of her RA. Based on limited evidence from case reports and the fact that these kinds of patients usually need simultaneous treatment for all of their conditions, outlining a clear association still represents a challenge.

## Figures and Tables

**Table 1:** Summary of reported cases and patient characteristics.

Case report	Race	Age	Sex	Other comorbidities	RF or ACPA	HLA type	Erosions	Other X-ray findings	Treatment received
Kawasaki et al. 2000 [6]	Japan	11	M	None	Negative	—	—	—	MTX, Prednisolone, and NSAIDS (doses not mentioned) and then later MTX changed to Bucillamine

Riente et al. 2001 [7]	Italy	60	F	DM, HTN, heart failure	Positive	A1, B44, B51, DRB1^*∗*^11 DRB1^*∗*^16 DRB3, DRB5	No	Symmetric narrowing of MCP + PIP with juxta-articular osteoporosis	Initially Chloroquine 500 mg/day orally + Methylprednisolone 4 mg/d Then Gold IM, low dose steroids + antibiotics

Rébora et al. 2006 [9]	Argentina	38	F	None	Positive	(i) A1, B8, B57 (ii) HLA DR not studied	Yes	Narrowing at the wrists + 3rd MCPs bilaterally with juxta-articular osteopenia	Initially SSZ 1.5 g per day + NSAIDS Later Chloroquine 200 mg and then HCQ 400 mg daily Subsequently shifted to MTX 15 mg/day

Younes et al. 2006 [8]	Tunisia	35	F	None	Positive	—	Yes	Narrowing at the MCPs + 2nd & 3rd PIPs	Indomethacin 100 mg daily, Prednisolone 10 mg orally daily and MTX 10 mg weekly later increased to 15 mg weekly

Takasaki et al. 2014 [10]	Japan	47	F	Periodontitis, smoker	Positive	—	Yes	Joint destruction in the RT thumb MCP + LT thumb MCP and PIP	MTX 12 mg weekly, Tacrolimus 2 mg/daily, Prednisone 4 mg daily Later shifted to Etanercept 50 mg/week + tacrolimus tapered off

*Our patient*	Saudi Arabia	18	F	—	Positive	—	No	Normal	MTX 12.5 mg PO weekly gradually increased until 20 mg weekly + Prednisolone 15 mg orally daily gradually tapered off

MTX = Methotrexate, HCQ = Hydroxychloroquine, and SSZ = sulfasalazine.
